# Uncovered self-expandable metal stents for the treatment of refractory benign colorectal anastomotic stricture

**DOI:** 10.1038/s41598-020-76779-8

**Published:** 2020-11-16

**Authors:** Ji Taek Hong, Tae Jun Kim, Sung Noh Hong, Young-Ho Kim, Dong Kyung Chang, Eun Ran Kim

**Affiliations:** 1 Department of Internal Medicine, Ewha Womans University College of Medicine, Seoul, Korea; 2 Division of Gastroenterology, Department of Medicine, Samsung Medical Center, Sungkyunkwan University School of Medicine, 81 Irwon-ro, Gangnam-gu, Seoul, 06351 Republic of Korea

**Keywords:** Colonoscopy, Colonic diseases, Colorectal cancer

## Abstract

Self-expandable metal stent (SEMS) placement has been suggested as a therapeutic modality for treating benign colorectal strictures. Covered stents are generally used, given the concerns regarding the efficacy and safety of uncovered stents. Hence, few studies have evaluated the efficacy and safety of uncovered SEMSs (UCSEMSs) in patients with refractory benign colorectal anastomotic strictures. In this study, 12 patients with postoperative benign symptomatic anastomotic strictures refractory to pneumatic dilation (range, 2–9) and transient indwelling-covered SEMSs were treated using UCSEMS. All enrolled patients were men (mean age, 61 years). Stent placement was successful in all 12 patients, and early clinical success was achieved in 11 (92%) patients. Four patients (25%) showed successful clinical outcomes without further intervention, but eight patients (75%) were clinically unsuccessful, and showed stricture recurrence or functional obstructive symptoms. Three patients underwent surgery, and the remaining five patients required repeat stent procedures. Despite the high reobstruction rate, the median follow-up period after UCSEMS placement was 16.7 months, demonstrating that UCSEMS may be able to achieve medium-term symptom relief without any complications. Therefore, UCSEMS may be an alternative option in exceptional circumstances in carefully selected patients, where invasive surgical treatments, such as stoma diversion, are not an option, thereby improving patients’ quality of life.

## Introduction

Benign colorectal obstruction can be caused by various factors related to diverticulosis, inflammation, ischemia, radiation, or anastomosis. Among these factors, anastomotic stricture is one of the most common complications of colorectal surgery^1^, with the incidence of anastomotic stricture or stenosis after colorectal surgery ranging between 0 and 30%^2–4^. Patients with clinically significant strictures typically exhibit partial or complete bowel obstruction.

Clinically significant colorectal, colocolic, or ileocolic strictures can be managed by minimally invasive methods, either endoscopically or radiologically. Endoscopic balloon dilation is reportedly successful in 88–100% of benign cases^2,5,6^, whereas endoscopic transanal resection of strictures has been described for managing high-grade anastomotic strictures^6^. However, commonly used endoscopic treatments are often ineffective due to the use of a balloon, and have a high recurrence rate of 30–88%, as well as refractoriness in > 20% of cases^6–10^, with such refractory strictures often requiring surgical revision.

Self-expandable metal stent (SEMS) placement is a widely used endoscopic treatment modality for strictures^11^. However, SEMS treatment was originally established to palliate malignant colorectal obstruction or as a bridge to surgery^12–15^. Recently, SEMS placement has also been suggested as a therapeutic modality for the relief of benign colorectal strictures. However, data regarding the use of SEMSs in patients with benign colorectal strictures have been obtained from heterogeneous studies, and the efficacy, safety, and long-term patency of SEMSs in these patients remains controversial^11,12,16–20^.

In addition, although both fully covered SEMS (FCSEMS) and uncovered SEMS (UCSEMS) have advantages and disadvantages, FCSEMSs are primarily used for benign strictures because of the reduced local tissue response^16^. To date, limited studies have examined the efficacy and safety of UCSEMS placement in patients with refractory benign colorectal strictures, and the limited available data have been obtained from a heterogeneous study^17^. Recently, biodegradable and drug-eluting stents have also been attempted or studied^21–25^. Despite their considerable advantages and good anecdotal results, their application in the large bowel is unlicensed, and further evidence is needed until they can be used widely. Furthermore, specific indications for covered and uncovered stents in patients with benign colorectal stenosis have not been well established^26,27^. Therefore, this study aimed to detail our experience with attempted UCSEMS placement for the treatment of refractory benign colorectal anastomotic strictures in a homogeneous group of patients, with the aim to offer an alternative treatment option and avoid invasive surgical treatment.

## Methods

This retrospective study included all patients treated for symptomatic postoperative colorectal anastomotic strictures at our institution. All patients were identified from the prospective database of our hospital. Between October 2012 and December 2017, 12 male patients (mean age: 61 ± 13.2 [43–89] years) with refractory benign colorectal anastomotic strictures underwent UCSEMS placement. The requirement for patient consent was waived given the retrospective nature of the study (approval ID: SMC2018-12-089).

**Figure 1:**
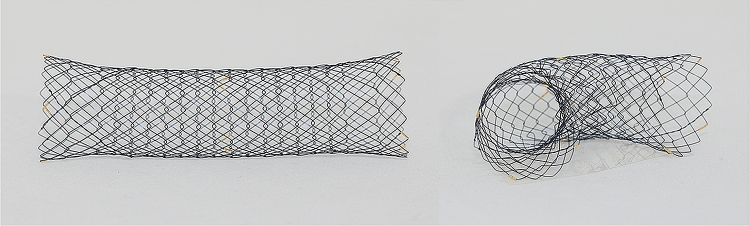
Uncovered self-expandable metal stents.

All endoscopic stent placements were performed under fluoroscopic guidance, and all stents were inserted by an experienced, board-certified interventional endoscopist (E.R.K.). The patients were placed in either the supine or left lateral decubitus position. Colon cleansing was achieved using oral polyethylene glycol and/or enemas. After visualization of the obstructed area with colonoscopy (12.2-mm outer diameter, Evis Lucera Colonovideoscope CF-H260AL/I; Olympus, Tokyo, Japan), a water-soluble contrast agent was injected to identify the stricture lesion and measure the length of the stricture. The length of the stent was chosen to cover the entire stricture lesion and extend beyond each end of the obstruction by at least 1–2 cm. Stenting was not performed when the stricture was located less than 4 cm from the anal verge (distal rectum), or when the stricture was > 8 cm. Once a guidewire (0.025-in. VisiGlide-2TM; Olympus Co., Tokyo, Japan) was positioned through the stricture area, a UCSEMS (length, 6 or 8 cm; diameter, 24 mm; BONA, Standard Sci Tech Inc., Seoul, South Korea or MI Tech, Seoul, South Korea) was placed (Fig. 1). Plain abdominal radiographs were obtained 24 h after the procedure to assess obstruction relief, as well as to confirm stent expansion and position.

Technical success was defined as adequate UCSEMS placement at the first attempt, with correct positioning and complete stent deployment at the stricture site confirmed using fluoroscopy. Early clinical success was defined as obstructive symptom relief and colorectal decompression persisting for a minimum of 3 days in the absence of endoscopic reinterventions or surgical procedures. Safety outcome was defined as the occurrence of complications; those leading to clinical failure, perforation, or a change in the treatment modality were labeled as major complications, whereas fecal incontinence, pain, foreign body sensation, hyperplastic tissue overgrowth, and self-limiting bleeding were labeled as minor complications. Early complications were defined as those occurring within 30 days after stent placement, whereas late complications were defined as those occurring over 30 days after stent placement.

Clinical failure was defined as the occurrence of obstructive signs and/or recurrence of symptoms any time after stent placement, which required reintervention or a surgical procedure. Stent migration was defined as either endoscopic visualization or radiographic evidence of stent movement from the initial position, total stent evacuation, or both. Clinical success was defined as persistent symptom improvement during follow-up, in the absence of reintervention or surgical treatment. The results are presented as mean (standard deviation) and median (range) unless otherwise stated. All analyses were performed using a statistical software package (SPSS 12.0 for windows, SPSS Inc., Chicago, IL, USA).

The study was approved by the Institutional Review Board of Samsung Medical Center (IRB No. SMC 2018-12-089), and the study was performed in accordance with the principles of the Declaration of Helsinki. The off-label indication of the procedure was fully explained to the patient, and written informed consent was obtained from all patients who received this treatment.

## Results

Twelve patients with benign colorectal strictures secondary to postoperative anastomotic strictures were treated using metal-uncovered stents between October 2012 and December 2017. All cases were refractory to at least two balloon dilations, and 6 of the 12 patients underwent covered SEMS insertion prior to UCSEMS insertion. In all cases, previous endoscopic dilations with and without covered SEMS for temporary drainage had been unsuccessful, with the number of dilations ranging from 2 to 9. The causes of treatment failure in the patients with a covered SEMS was anal pain caused by stent movement (2/6, 33%) and stent migration (4/6, 67%). All patients had symptoms of intestinal obstruction 24–48 h before UCSEMS insertion. Six patients (50%) had complete obstruction, confirmed fluoroscopically using water-soluble contrast agent injection, and endoscopically through direct visualization. Histological analysis confirmed that all strictures were benign.

The baseline characteristics of the patients are presented in Table 1. The etiology of the obstruction was postsurgical anastomotic strictures in all patients. Six patients with rectal cancer underwent lower anterior resection with protective ileostomy, one patient with sigmoid colon cancer underwent the Hartmann procedure, one patient with rectal cancer underwent lower anterior resection without protective ileostomy, three patients with sigmoid colon cancer underwent laparoscopic anterior resection, and one patient with a pancreatic pseudocyst underwent right colectomy. Moreover, history of neoadjuvant chemoradiotherapy (*n* = 5), adjuvant chemotherapy (*n* = 5)*,* and adjuvant chemoradiotherapy (*n* = 1) was observed.

**Table 1:** Characteristics of patients undergoing uncovered metal stenting for benign anastomosis strictures.

Sex/age (years)	Etiology	Surgery	Preoperative therapy	Postoperative therapy	Degree of obstruction	Previous endoscopic treatment	Diverticular disease
M/57	Rectal Ca	SILS-LAR c protective ileostomy	Neoadjuvant CCRT	Adjuvant CT	Total	Balloon dilations + FCSEMS (22 days: removal due to anal pain caused by stent movement)	No
M/66	Rectal Ca	LAR	–	Adjuvant CCRT	Total	Balloon dilations	No
M/58	Rectal Ca	HALS-LAR c protective ileostomy	Neoadjuvant CCRT	Adjuvant CT	Subtotal	Balloon dilations + FCSEMS (14 days: stent migration)	No
M/73	Rectal Ca	LAR c protective ileostomy	Neoadjuvant CCRT	–	Subtotal	Balloon dilations	No
M/55	Rectal Ca	LAR c protective ileostomy	–	Adjuvant CT Palliative CT	Total	Balloon dilations + FCSEMS (15 days: removal due to anal pain caused by stent movement)	No
M/57	Sigmoid colon Ca	AR	Neoadjuvant CCRT	–	Subtotal	Balloon dilations	No
M/43	Pancreatitis, pseudocyst	Rt colectomy c ileostomy	–	–	Total	Balloon dilations	No
M/89	Sigmoid colon Ca	AR	–	–	Total	Balloon dilations	No
M/45	Rectosigmoid colon Ca	SILS-LAR c protective ileostomy	Neoadjuvant CCRT	–	Subtotal	Balloon dilations + FCSEMS (2 days: stent migration)	No
M/51	Rectosigmoid colon Ca	Hartmann operation	–	–	Subtotal	Balloon dilations + FCSEMS (47 days: stent migration)	No
M/70	Rectosigmoid colon Ca	SILS-AR c protective ileostomy	–	Adjuvant CT	Total	Balloon dilations	Yes
M/72	Rectal Ca	LAR c protective ileostomy	–	–	Subtotal	Balloon dilations + FCSEMS (14 days: stent migration)	Yes

Ca.: cancer, SILS: single-incision laparoscopic surgery, LAR: low anterior resection, HALS: hand-assisted laparoscopic surgery, AR: anterior resection, CCRT: concurrent chemoradiotherapy, CT: chemotherapy, FCSEMS: fully covered self-expandable metal stent; c, with.

**Figure 2:**
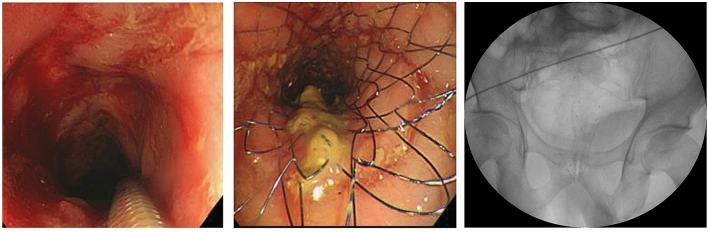
Endoscopic and fluoroscopic images showing successful insertion of an uncovered self-expandable metal stent for refractory benign colorectal anastomotic stricture.

All 12 patients (100%) achieved technical success (Fig. 2) and underwent intraprocedural decompression immediately following stent insertion. Clinically and radiologically, UCSEMS successfully relieved obstruction in 11 patients (92%). Early clinical success was achieved in 11 of the 12 (92%) cases (Table 2). Subsequently, the patients underwent clinical and endoscopic follow-up, with a median follow-up period of 16.7 (1.7–35.37) months after UCSEMS placement. The follow-up period and outcomes for each patient are detailed in Table 3.

**Table 2:** Indications for UCSEMS insertion and early clinical outcomes.

Sex/age (years)	Reason for considering UCSEMS insertion	Technical success	Early clinical success	Distance from AV (cm)	Stent length (cm)	Stent diameter (mm)
M/57	FCSEMS failure	Yes	Yes	5	6	24
M/66	Poor candidate for surgery	Yes	Yes	10	6	24
M/58	FCSEMS failure	Yes	Yes	8	6	24
M/73	Poor candidate for surgery	Yes	Yes	7	6	24
M/55	FCSEMS failure	Yes	Yes	7	6	24
M/57	Refusal of surgical treatment	Yes	Yes	18	6	24
M/43	Refusal of surgical treatment	Yes	Yes	45	8	24
M/89	Poor candidate for surgery	Yes	Yes	15	6	24
M/45	FCSEMS failure	Yes	No	5	6	24
M/51	FCSEMS failure	Yes	Yes	12	4	24
M/70	Poor candidate for surgery	Yes	Yes	17	12	24
M/72	FCSEMS failure, poor candidate for surgery	Yes	Yes	8	6	24

AV: anal verge, US: uncovered stent, CS: covered stent, TH: tissue hyperplasia.

**Table 3:** Stent placement follow-up outcomes.

Sex/age (years)	Duration of luminal patency (days)	Stent outcomes	Endoscopic reintervention (time after stent insertion) (days)	Follow-up outcome (time after stent insertion)	Clinical failure	Clinical success	Operation	Stoma
M/57	1047	1st: obstruction	Restenting (1047)	No operation; AS at day 1047	Yes	No	NA	NA
M/66	284 (1st US), 777 (2nd US)	1st: obstruction, broken S	Restenting (284)	No operation; AS at day 284 and 777	Yes	No	NA	NA
M/58	685 (1st US), > 225 (2nd US)	1st: obstruction	Restenting (685)	No operation; AS at day 685 and 225	Yes	No	NA	NA
M/73	21(1st US), > 169 (2nd US)	1st: migration	Restenting (21)	Two-stage (day 190)	Yes	No	Transverse colon loop colostomy	Yes
M/55	155 (1st US), 155 (2nd CS), 192 (3rd CS), (4th CS)	1st: obstruction, TH 2nd: migration 3rd: anal pain	Restenting (155)	No operation; AS at day 155, 155, and 256	Yes	No	NA	NA
M/57	> 197	–	N	No operation; AS at day 197	No	Yes	NA	NA
M/43	399 (1st US), > 11 (2nd CS)	1st: obstruction, TH	Restenting (399)	Single-stage (day 410)	Yes	No	Segmental resection with primary anastomosis	No
M/89	> 51	–	N	No operation; AS at day 51	No	Yes	NA	NA
M/45	2 (1st US), 2 (2nd CS), 2 (3rd US)	1st, 2nd, 3rd: stent migration	Restenting (2)	Two-stage (day 6)	Yes	No	Transverse colon loop colostomy	Yes
M/51	> 180	–	N	No operation; AS at day 180	No	Yes	NA	No
M/70	196 (1st US), 12 (2nd US), 95 (3rd US), > 757 (4th US)	1st: obstruction, TH 2nd, 3rd: obstruction	Restenting (196)	No operation; AS at day 196, 12, 95, and 757	Yes	No	NA	No
M/72	> 574	–	N	No operation; AS at day 574	No	Yes	NA	No

AS: asymptomatic, S: stent, N: none, NA: not applicable, Two-stage: colostomy/ileostomy with stoma, Single-stage: colectomy w/1° anastomosis.

The outcomes of UCSEMS insertion were determined in all patients. In particular, among the 12 patients, 3 (25%) underwent surgery and 4 (33%) achieved clinical success without further intervention during a median follow-up period of 16.7 months. The remaining 5 patients required repeat stent procedures.

Migration was observed in 2 (17%) of the 12 patients within the first 30 days. All instances of stent migration occurred in patients with partial intestinal obstruction from the time of diagnosis.

**Figure 3:**
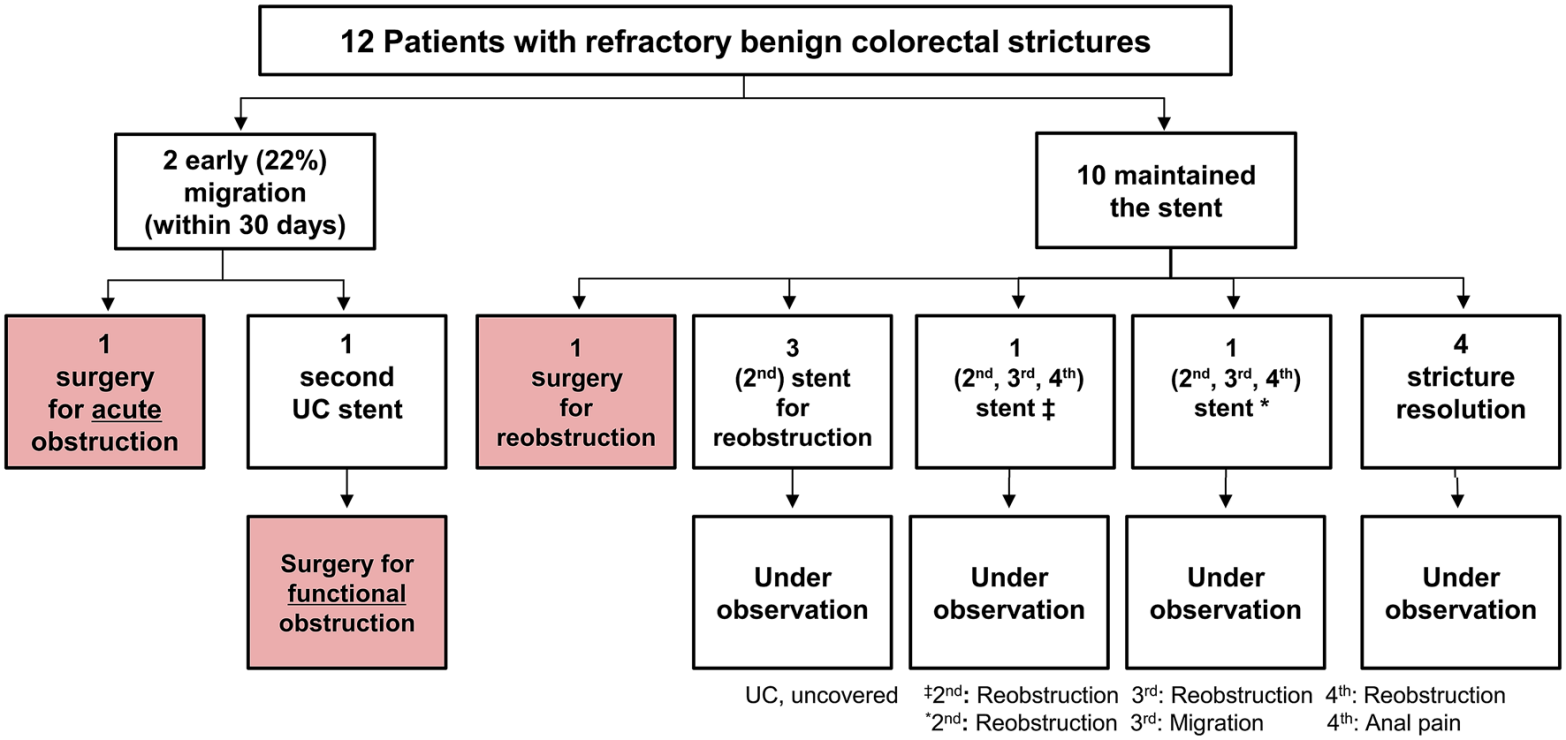
Study flowchart.

Reobstruction occurred in six patients (50%) (mean period: 461 days [range: 155–1047 days]), and multiple additional stents were reinserted after reobstruction. Of these six patients, five who underwent restenting remained under observation at the outpatient clinic without recurrence or complications. One patient initially underwent restenting but failed to maintain endoscopic stent patency and exhibited worsening of symptoms, for which elective surgical segmental resection was performed. A total of 9 SEMS reinsertions were performed in 5 of the 11 patients who maintained endoscopic stent patency as a result of restenosis (tissue ingrowth through the mesh) (Fig. 3). All re-occlusions were the result of epithelial hyperplasia, mucosal edema, and/or transmural fibrosis.

No major UCSEMS-related complications, such as perforation, occurred throughout the study period. Minor complications were observed in 3 of the 12 patients (25%); one exhibited stent fracture and the other two exhibited hyperplastic tissue overgrowth. Stent fracture was incidentally noted during regular follow-up endoscopic examinations without any specific symptoms. At the median follow-up period of 16.7 months, 4 (25%) of the 12 patients achieved clinical success.

**Figure 4:**
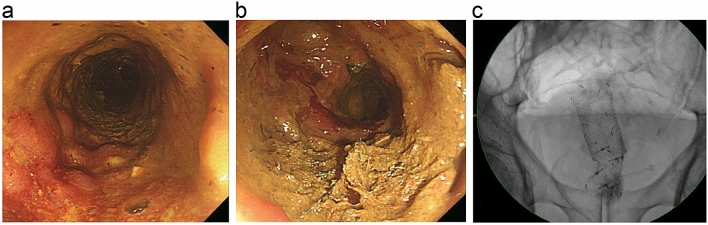
Patient with endoscopic stent patency who underwent surgery due to worsening obstructive symptoms. (**a**) Successful insertion of the second uncovered stent after the migration of the first stent. (**b**,**c**) Stent patency maintained in the endoscopy that was re-performed due to worsening obstructive symptoms.

Finally, three patients underwent surgery, one of whom failed to maintain endoscopic stent patency because of restenosis, and the two remaining patients experienced persistent functional obstruction despite successful additional stent replacement (median, 2.9; range, 6 days–5.6 months). Two patients eventually underwent surgery because of stent functional failure (Fig. 4).

## Discussion

The use of SEMS for benign strictures remains controversial. Accordingly, SEMSs used for benign colorectal diseases are associated with higher rates of stent-related complications, such as perforation, stent migration, recurrent obstruction, bleeding, and mucosal overgrowth than those used for malignant diseases^11–13,17,28–30^. Although concerns regarding efficacy and safety have previously limited the use of SEMs, their use for benign diseases has been attempted^18,20,31^.

Covered stents are generally used for benign gastroduodenal or colorectal strictures, considering that uncovered stents may cause tissue embedding through the stent’s metal meshwork during the follow-up period. In addition, UCSEMS cannot be removed; therefore, their use has always been intended as a bridge therapy to surgery or as a palliative therapy in patients with malignant strictures. However, covered stents have a considerable advantage in preventing granulation tissue formation^32,33^. Moreover, reports showing significant morbidity in patients with benign strictures who underwent UCSEMS placement have further limited their use. As a result, there have been few attempts to use uncovered stents for the treatment of benign strictures, and related reports and follow-up data have been markedly limited.

The present study investigated the efficacy and safety of UCSEMSs for refractory post-anastomotic strictures in a highly select patient group. Among the strengths of this study, the first is that it examined stent efficacy in a single group of patients in whom obstruction had been caused by a post-anastomotic stricture. Second, it included a refractory group of patients who were not responsive to conventional endoscopic treatment. Finally, we investigated the effectiveness and safety of UCSEMS placement in the aforementioned group. To the best of our knowledge, this is the first study to attempt UCSEMS placement in a homogeneous group of patients with refractory anastomotic strictures.

The effectiveness of SEMSs has mostly been dependent on the etiology of the stricture^16,18,29,34–36^. Stents have been effective in cases of postsurgical colorectal strictures, whereas they have been deemed inappropriate in cases of ischemic strictures, diverticular strictures, and Crohn’s disease^20^. Therefore, evaluating the effects of stents on strictures in a homogeneous group with a similar etiology is imperative. However, most studies on benign colorectal strictures involve a variety of etiologies.

Benign anastomotic strictures of the rectosigmoid colon that fail to respond to repeated endoscopic dilation attempts may represent potential indications for stent placement. Although biodegradable and drug-eluting stents have also been recently studied, or their placement has been attempted, they have not shown satisfactory results, and information regarding their use in the lower gastrointestinal tract has remained limited^21–25,37^. Despite their considerable advantages, current biodegradable stents have not yet been widely used, and access to these stents is limited in many countries. Furthermore, biodegradable stents specially designed for colonic strictures are not yet available. In cases where treatment with stents for benign colorectal strictures fails, other investigational or surgical treatments are considered^27^. However, surgical revision, such as stoma diversion, may be very difficult because of adhesion, stricture, and local inflammation frequently located in the lower rectum, and the results are often suboptimal, particularly in emergency situations^4^. Moreover, specific indications for FCSEMSs and UCSEMSs in benign colorectal stenosis have not been well established. Therefore, although UCSEMS can no longer be endoscopically removed, and there are limited data on the real risk of maintaining a UCSEMS in the colon for a prolonged period, the inclusion of patients with refractory colorectal stricture in the present study is of significant importance. This is particularly relevant given that UCSEMSs offer an alternative option, and help avoid invasive surgical treatments for patients who are not suitable for surgery because of morbidity or adhesion^38^.

Small et al. reported complications, including migration, reobstruction, and perforation, in 38% (9/23) of cases involving benign colorectal strictures that were treated with uncovered stent insertion^17^. For this reason, FCSEMSs have been primarily used for benign strictures. However, data regarding the safety and efficacy of UCSEMSs have been obtained from patient groups with heterogeneous etiology. Among the 23 patients included in the study conducted by Small et al., only three had postsurgical anastomotic strictures. Furthermore, postoperative strictures caused complications in only one case, with migration being the major complication, while the remaining two patients were asymptomatic for 1 and 10 months without complications. Moreover, the median follow-up period was only 6 months. Other studies have also revealed relatively favorable results for SEMS among patients with post-anastomotic strictures^20,35^.

Studies of FCSEMS in benign anastomotic strictures have shown a clinical success rate of approximately 36–71.4%, with most complications being caused by migration^19,39–41^. UCSEMSs have a lower rate of migration than covered stents; however, compared with covered stents, uncovered stents are subject to tissue hyperplasia when used for a prolonged period of time. Uncovered stents are normally impossible to remove because of the hyperplastic reaction, and can only be removed surgically. Because of these disadvantages, uncovered stents have not been used for benign strictures given concerns regarding morbidity. Finally, surgical treatment may be required for patients who fail endoscopic remediation. However, for patients who are considered to be even unfit to undergo surgery, other treatment options will have to be explored. Therefore, it is also necessary to examine the effectiveness and safety of UCSEMSs in patients with post-anastomosis strictures who showed relatively favorable outcomes after stent treatment in previous studies. As yet, there has been a paucity of studies on this topic; nonetheless, the significant findings of the present study suggest that UCSEMSs are a potential single-treatment option in specific groups.

Other significant findings presented herein include follow-up data on UCSEMSs for refractory benign anastomotic colorectal strictures. In one case report of a patient with a benign colorectal stricture, Monzur et al. revealed that long-term UCSEMS placement proved to be a reliable treatment option for over 4 years^1^. The clinical course of the patient was complicated by recurrent episodes of hematochezia, which is consistent with the increased risk of gastrointestinal bleeding associated with prolonged SEMS use. However, no UCSEMS-related bleeding was observed in our study, and technical success was achieved in all cases. The early clinical success rate was also high, with relief of obstructive symptoms observed in > 92% of the patients. These results are noteworthy given that in all patients in the study population, there was failure to obtain temporary drainage using covered SEMSs or endoscopic balloon dilations, and such cases would typically be difficult to manage. Under these circumstances, such refractory strictures may require surgical revision. However, if other endoscopic treatments, such as covered, removable, or biodegradable stents, fail, and if the patient is not fit to undergo surgery, UCSEMS placement may be considered to avoid further surgical treatment in the short term. Accordingly, our results suggest that UCSEMS placement for the treatment of refractory benign colorectal anastomotic strictures may be considered in selected patients. However, the limited number of patients included in this study, as well as the study design, may prevent us from drawing definitive conclusions. At the median follow-up period of 16.7 months, only 4 (25%) of the 12 patients had achieved clinical success. As expected, the reobstruction rate was high, with stent reobstruction occurring in > 50% of the patients. Nonetheless, our experience suggests that the duration of stent placement was sufficient to attain a clinical medium-term symptom resolution (461 [range: 155–1047] days). These outcomes show that UCSEMSs can be used as a bridge to therapy preoperatively, or as a palliative therapy in patients unfit for surgery if other endoscopic treatments are found to be ineffective against refractory benign colorectal strictures. Even if reobstruction occurs, the stent can be reinserted to alleviate the symptoms and maintain stent patency.

Considering that the long-term safety and efficacy of UCSEMSs as a permanent therapy remains to be determined, data for long-term UCSEMS placement are still unavailable. More research is required to validate the long-term safety and efficacy of UCSEMSs in refractory benign colorectal strictures.

In the present study, two patients who underwent UCSEMS placement exhibited stent migration. In one case, the patient had undergone balloon dilation nine times, and endoscopic transanal resection once prior to stent placement. All instances of stent migration occurred in cases with partial intestinal obstruction since the time of diagnosis, wherein obstructive symptoms might have resulted from a nonfunctioning bowel rather than from an obstruction. In such cases, the prognosis of stent insertion was projected to be poor. Therefore, surgical treatment can be considered from the very beginning in patients with refractory anastomotic strictures due to partial obstruction.

Perforation, which remains one of the most serious complications of stent placement, usually occurs immediately after stent placement. Moreover, rapid stent expansion, balloon predilatation, and excessive stricture manipulation have been shown to increase the risk of perforation^42–44^, with radiation-induced strictures being particularly vulnerable to injury. The high incidence of complications seems to be related to the aforementioned factors^35,45^. Although the patients in the current study displayed some risk factors, such as radiation and balloon predilatation, no UCSEMS-related perforation occurred.

The present study has limitations such as the small sample size (21 procedures in 12 patients) and the retrospective design. Moreover, the rate of complications in the present study was high (75%). Even though data were prospectively collected, the study was performed retrospectively with no control group. However, no comparable standard treatment exists for refractory colorectal strictures through which a control group can be established, and recruitment limitations led to an insufficient number of patients for a prospective randomized trial in a single-center setting.

Despite these limitations, among similar studies conducted to date, this study included the largest homogeneous group of patients with refractory benign anastomotic strictures to evaluate the effectiveness and safety of UCSEMS placement. Follow-up data concerning UCSEMS placement in patients with benign refractory strictures are very limited, with no follow-up being performed for most cases after UCSEMS placement. The median follow-up period after UCSEMS placement was 16.7 (range, 1.7–35.37) months in this study. With a medium-term follow-up period, this study provides a unique perspective on the efficacy of this endoscopic option over time.

In conclusion, UCSEMS placement for symptomatic refractory benign anastomotic strictures may be selected on an individual basis, taking into account the comorbidity, estimated life expectancy of the patient, or before permanent colostomy for patients who have contraindications to or refuse permanent colostomy. Furthermore, patients should be counseled very carefully regarding the likely necessity of repeating the procedures. However, despite the high reobstruction rate, UCSEMS may be able to achieve uncomplicated medium-term symptom relief and avoid more invasive surgical treatments, such as stoma diversion, thereby improving patients’ quality of life. Prior to surgical or experimental approaches, UCSEMS placement may be considered as a treatment option for post-anastomotic strictures in selected patients who are non-responsive to conventional endoscopic treatments and are considered unfit to undergo surgery. Surgical treatment may be considered from the beginning in patients in whom post-anastomotic strictures remain unresponsive to endoscopic treatment owing to partial obstruction, given that their symptoms are attributable to a nonfunctioning bowel. Our experience verifies that UCSEMS may be used as a last resort treatment option in patients with an otherwise poor prognosis, or who have no alternatives.
